# Anterior Closing-Wedge High Tibial Osteotomy Using Patient-Specific Cutting Guide in Chronic Anterior Cruciate Ligament–Deficient Knees

**DOI:** 10.1016/j.eats.2022.05.006

**Published:** 2022-09-21

**Authors:** Sylvain Guy, Raghbir Khakha, Matthieu Ollivier

**Affiliations:** a Institute of Movement and Locomotion, Department of Orthopedics and Traumatology, St. Marguerite Hospital, Marseille, France; b Institute for Locomotion, Aix Marseille University, APHM, CNRS, ISM, Marseille, France; c Guy’s and St. Thomas’ Hospitals, London, United Kingdom

## Abstract

An increased posterior tibial slope has been identified as an independent risk factor for anterior cruciate ligament (ACL) graft rupture, with a critical threshold of 12°. Surgical slope correction by anterior closing wedge (ACW)-high tibial osteotomy (HTO) can reduce ACL force and anterior tibial translation with good clinical outcomes when combined with revision ACL reconstruction. Performing ACW-HTO preserving the tibial tubercule can be challenging for inexperienced surgeons. Patient-specific cutting guides have been shown to be effective in facilitating the surgeon's learning curve in medial opening wedge-HTO by reducing operative time and the use of fluoroscopy as well as decreasing anxiety. The present technique describes a retro-tibial tubercule ACW-HTO using a patient-specific cutting guide.

An increased posterior tibial slope (PTS) is a well-described anatomical risk factor for anterior cruciate ligament (ACL) graft rupture.^1^, ^2^, ^3^, ^4^ Webb et al. described a threshold of 12°, after which a patient undergoing primary anterior cruciate ligament reconstruction (ACLR) has a 4.52- to 5.2-fold increased risk of graft rupture^5^^,^^6^ and up to 11-fold in adolescents.^7^ Cadaveric studies have demonstrated the direct correlation between an increased PTS steepness and ACL stress, intensified by sporting activities.^8^^,^^9^ Surgical slope adjustment by anterior-closing wedge (ACW)-high tibial osteotomy (HTO) has been shown reduced ACL force and anterior tibial translation in vitro.^10^^,^^11^ These results have been confirmed in small clinical series, which have reported good patient-reported outcomes with no graft rerupture at 2 years’ follow-up, after combined slope correction osteotomy and revision ACLR.^12^, ^13^, ^14^

Some authors describe ACW-HTO techniques preserving the tibial tubercle (TT), thus maintaining the integrity of the extensor mechanism. The osteotomy can be performed distal to the TT, but the resulting high distraction stress of the extensor mechanism may adversely increase the risk of pseudoarthrosis.^15^^,^^16^ Queiros et al.^17^ avoid this pitfall by performing a retro-TT osteotomy, which can be technically challenging. Patient-specific cutting guides (PSCGs) have proven to be effective in facilitating the surgeon's learning curve in medial opening wedge (MOW)-HTO by reducing operative time and the use of fluoroscopy as well as decreasing anxiety.^18^ The PSCGs are designed based on a preoperative computed tomography (CT) scan, so they allow for a precise correction in accordance with the surgeon's aims.^19^^,^^20^ No study to date has explored the use of PSCGs and their value in ACW-HTO. We anticipate they could facilitate the surgical procedure and improve reproducibility for less experienced surgeons. The present technique describes a retro-TT ACW-HTO using a PSCG (Table 1, Video 1).

**Table 1:** Surgical Steps, Pearls, and Pitfalls of the Present Technique

Surgical Step	Pearls	Pitfalls and Tips to Avoid Them
• Proximal medial tibia exposure	• Careful subperiosteal detachment of the medial collateral ligament allows exposure of the medial aspect of the proximal tibia without damaging the MCL.	• The detachment must be performed to the most posterior aspect of the proximal medial tibia to correctly position the cutting guide.
• Proximal lateral tibia exposure	• Subperiosteal detachment of the TA should be performed starting from its most medial part working round to the lateral aspect.	• Enough fascial tissue must be preserved medially and proximally to allow the TA to be properly reattached at the end of the procedure.
• Identification and dissection of the patellar tendon	• The medial and lateral edges of the patellar tendon must be dissected so that the proximal connector of both PSCGs can be passed back behind it.	• Improper dissection of the patellar tendon could result in damage to the latter during passage of the connector and performance of the osteotomy. Its lateral and medial edges can be individualized with the monopolar diathermy.
• Identification of the TT	• The TT must be identified to safely perform the vertical osteotomy aimed at preserving it.	• Incomplete identification of the TT would be at risk of iatrogenic fracture thus damage to the extensor mechanism.
• Positioning of the PSCGs and K-wires, drilling of the screw holes	• The PSCGs must be positioned exactly as planned on the tibial cortex and secured with K-wires. Peroperative fluoroscopy allows the correct position of the guides and K-wires to be checked.	• The fluoroscopy device must be positioned to get a true lateral view of the knee defined by the superposition of the femoral condyles. Incorrect positioning could mislead the surgeon as to the correct placement of the PCSGs and K-wires, and thus the incorrect performance of the osteotomy.
• Performing the osteotomy: 1 vertical cut behind the TT and 4 anteroposterior cuts	• The first cut must be the vertical cut behind the TT. The 4 anteroposterior cuts must be performed with an oscillating saw guided by the PSCGs until the protective K-wires are reached.	• The saw must not force on the cutting guide. The anteroposterior cuts must stop at the protective posterior K-wires and can be controlled by fluoroscopy. The length of the saw to be driven into the tibia can be calculated on the pre-operative planning and carried over intraoperatively to improve the safety of the procedure.
• Anterior bone wedge removal, reduction, and fixation	• The K-wires and PSCGs are pulled out. Once the anterior bone wedge is removed, reduction should be performed with the knee in full extension. Osteosynthesis must be performed by positioning the plate in line with the screw holes previously drilled.	• To prevent posterior hinge fracture, the protective posterior K-wires must be left in place during reduction and plate fixation.

MCL, medial collateral ligament; PSCGs, patient-specific cutting guide; TA, tibialis anterior; TT, tibial tubercle.

## Surgical Technique (With Video Illustration)

Our indication for ACW-HTO surgery is based on patients with chronic anterior instability of the knee with at least a second ACL graft rerupture (Table 2). The diagnosis of ACL graft rupture is based on magnetic resonance imaging and a complete clinical examination. Surgical management is decided upon when the patient’s subjective instability restricts them from sports or daily activities. The posterior tibial slope is determined on preoperative lateral knee radiographs as the angle between the proximal anatomic axis of the tibia and the tangential line to the medial tibial plateau.^5^ If this exceeds 12°, the patient is counseled regarding the option of a slope correction osteotomy combined with a revision ACLR.^5^, ^6^, ^7^

**Table 2:** Indications and Contraindications of the Present Technique

Indications	Contraindications
• Two or more ACL graft ruptures • Preoperative PTS >12° • Restraining ACL-related instability	• Preoperative recurvatum >5° • History of TT osteotomy • Symptomatic PCL-related instability

ACL, anterior cruciate ligament; PCL, posterior cruciate ligament; PTS, posterior tibial slope; TT, tibial tubercle.

### Preoperative Planning

An automated osteotomy planning software (PeekMed, Braga, Portugal) is used to define the amount of correction of the slope. The postoperative target is defined based on the amount of excessive tibial slope with the aim to correct to 5 to 7° PTS after surgery.^21^

A CT scan protocol is used to create the PSCG, consisting of images centered on the femoral head, the knee (allowing the distal femur and 15 cm of the proximal tibia to be captured), and slices centered over the ankle joint. The slice thickness is 0.625 mm for the knee and 2 mm for the hip and ankle (GE LightSpeed VCT64; GE, Milwaukee, WI). From the acquired images, a 3-dimensional (3D) model of the tibia is created. The desired slope correction is simulated using these images to match the desired correction in the sagittal plane defined by the surgeon.

**Fig 1:**
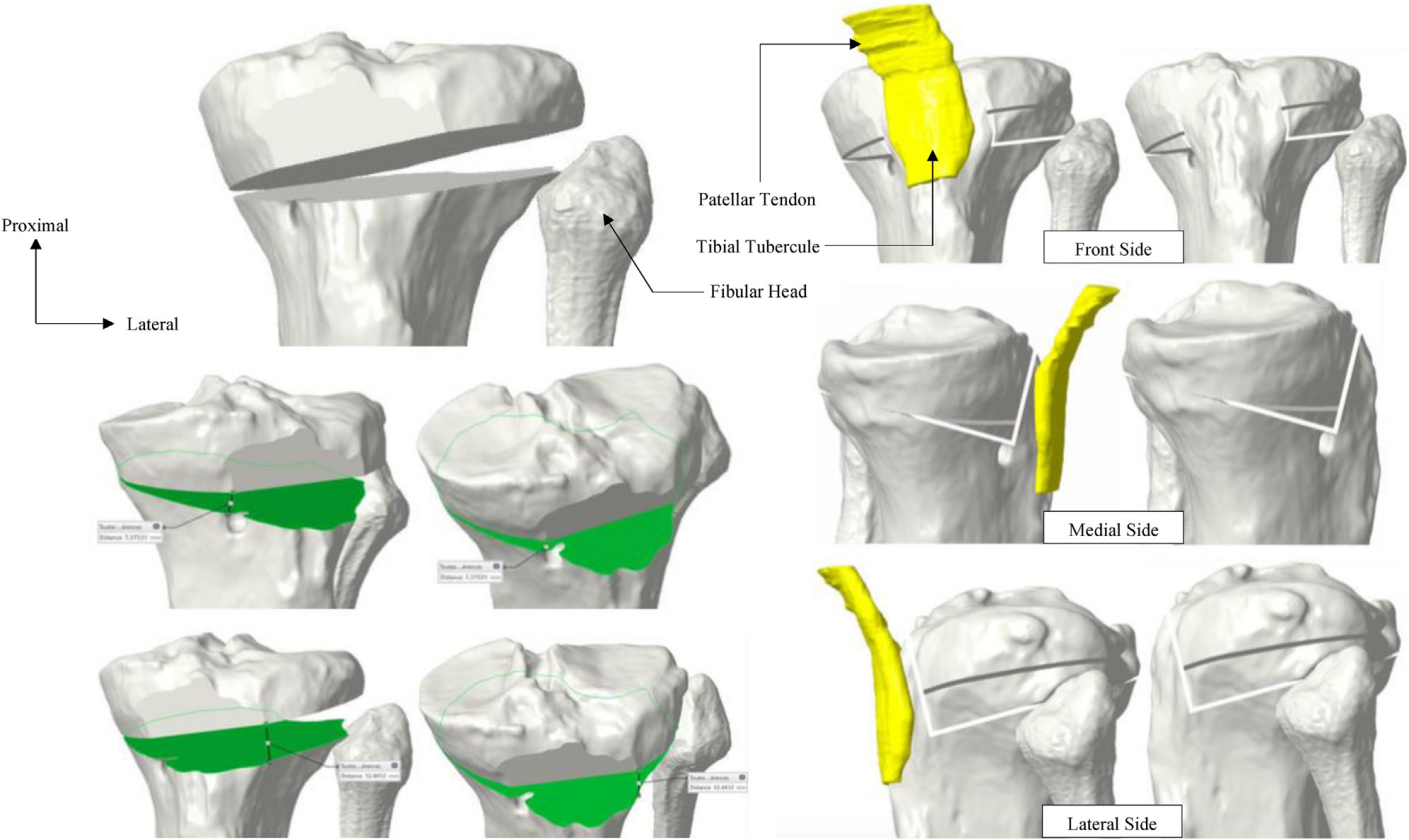
Simulation of the biplanar osteotomy on the preoperative computed tomography scan. The desired correction for this patient was 8° in the frontal plane (increase in medial proximal tibial angle) and 6° in the sagittal plane (decrease in posterior tibial slope). The cut is therefore asymmetrical at the expense of the lateral side to correct the posterior slope and the varus simultaneously.

After simulating the osteotomy on the 3D tibial model (Fig 1), the plate (Activmotion closing wedge plate, Newclip Technics, Haute-Goulaine, France) is virtually positioned to stabilize the osteotomy. The screws placement and sizes are also calculated. Once the final construct is virtually defined, the preosteotomy cutting jig is designed to guide the cut and to drill the final screws holes.

### Patient Setup

The patient is positioned supine on a standard operative table. A foot roll is positioned to hold the knee at 90° of flexion, with an additional lateral post proximal to the knee preventing external hip rotation. Fluoroscopy is positioned such that a true lateral view of the knee can be obtained intraoperatively, defined by the superposition of the femoral condyles. A tourniquet is inflated throughout the procedure.

### Surgical Procedure

**Fig 2:**
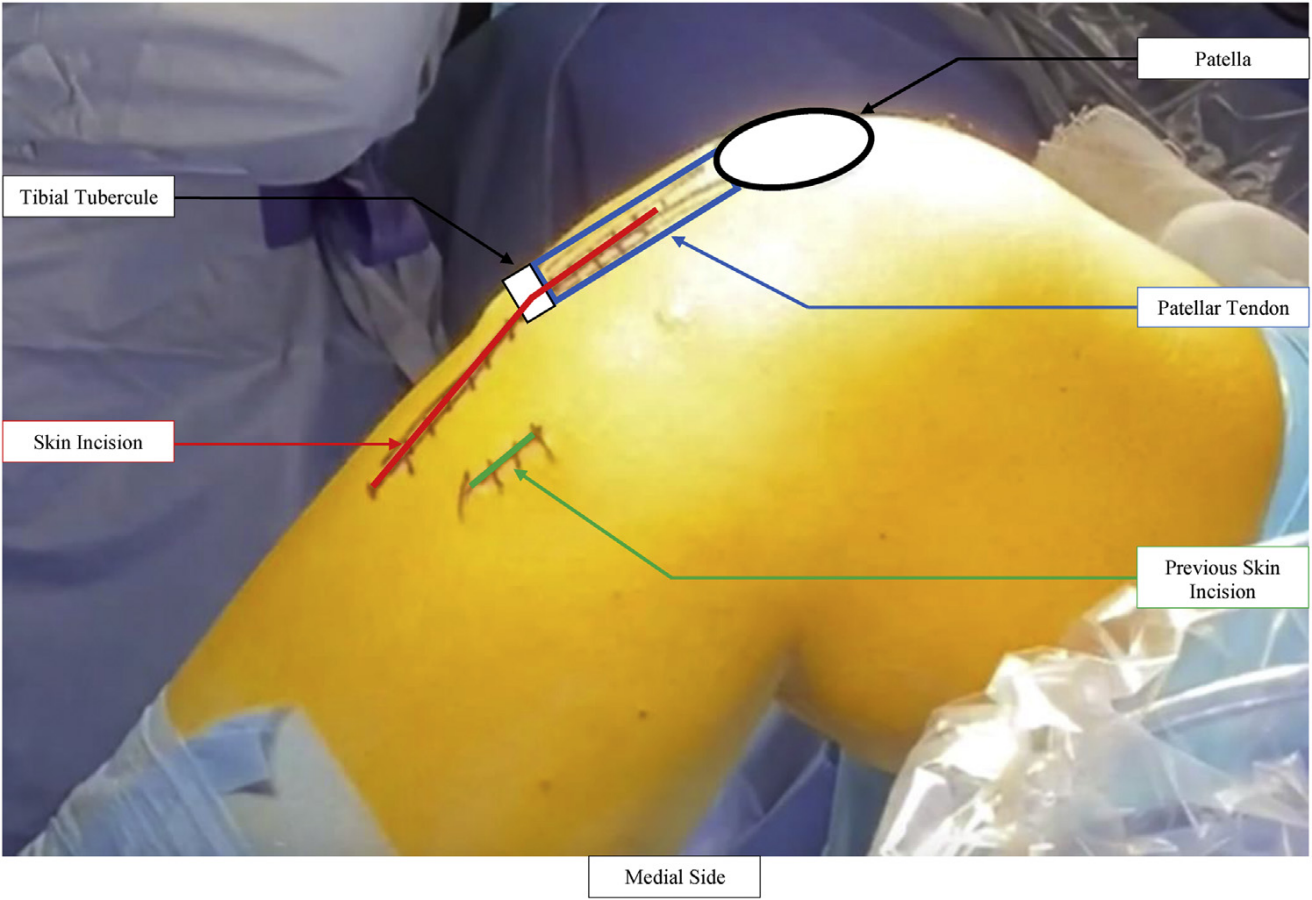
The knee is positioned at 90° of flexion. Careful attention must be paid to the previous incisions which must be marked. The patella, the patellar tendon, and the tibial tubercle are identified: the incision is longitudinal and centered on these landmarks.

**Fig 3:**
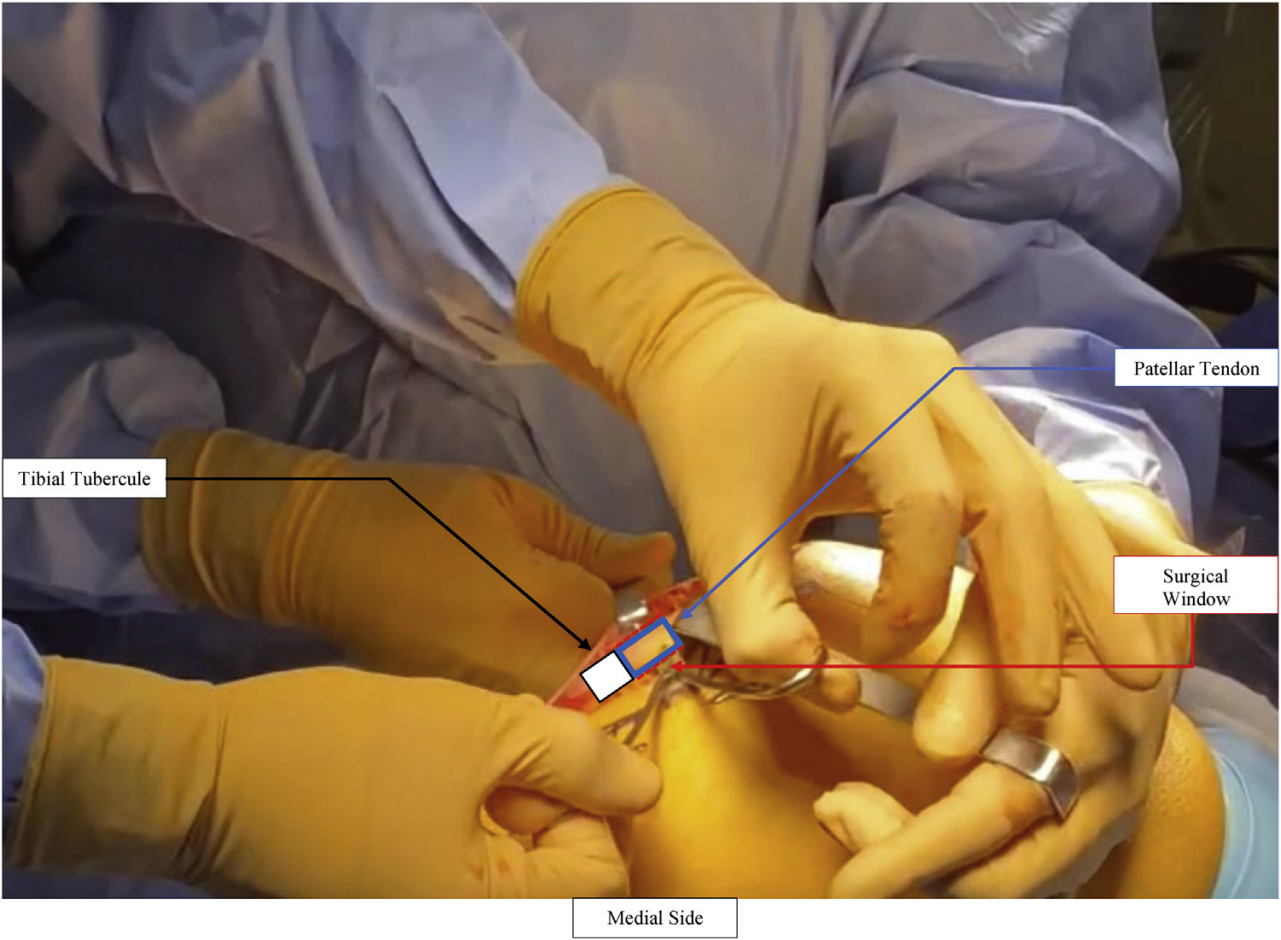
The patellar tendon is identified and dissected. A small window is created behind the patellar tendon just above the tibial tubercule to facilitate the future insertion of the proximal connect.

The anterior part of the proximal tibia is exposed through anterior longitudinal incision centered on the tibial tubercle (Fig 2). Medial dissection involves subperiosteal detachment of the deep medial collateral ligament. Lateral exposure is allowed by release of the tibialis anterior from its proximal attachment. A small window is created behind the patellar tendon and above the tibial tubercle (Fig 3).

**Fig 4:**
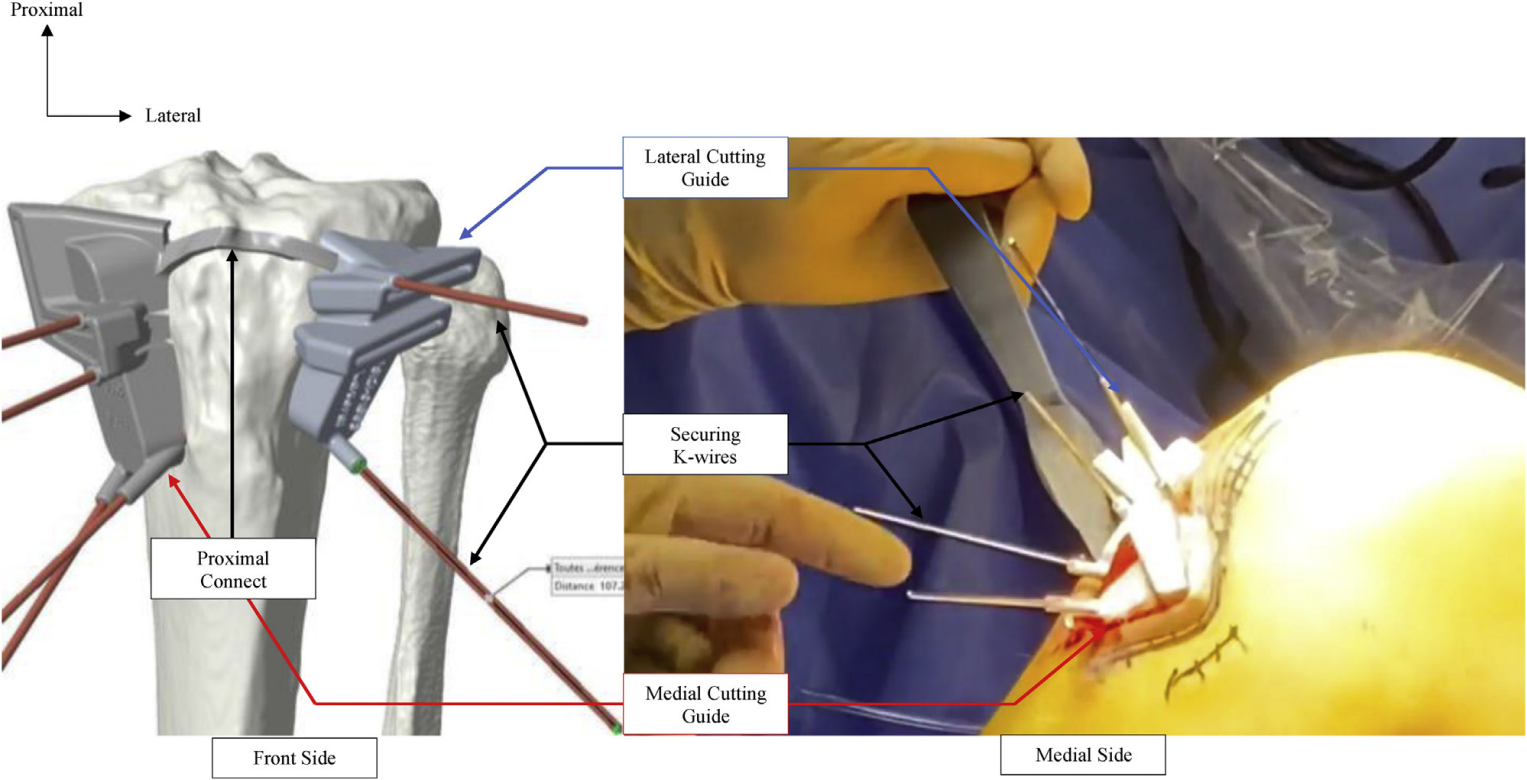
The medial and lateral patient-specific cutting guide are positioned and secured with K-wires. The proximal connect is positioned deep to the patellar tendon through the surgical window.

The 2 cutting guides are then positioned and connected to the tibia using the proximal connect, which sat deep to the patellar tendon through this window. Once the 2 guides are adequately positioned onto the medial and lateral side of the tibial tuberosity, 4 K-wires are introduced to guide the saw inside anteroposterior windows and 2 further K-wires are positioned to protect the posterior hinge from the saw (Fig 4). We then drill the 4 medial “screw holes” and secure the medial cutting guide using 4-mm K-wires.

To avoid detachment of the extensor mechanism, a first vertical cut in the sagittal plane is performed just posterior to the TT keeping its distal portion intact. The 4 different anteroposterior cuts are performed until the saw reaches the posterior hinge protecting k-wires.

**Fig 5:**
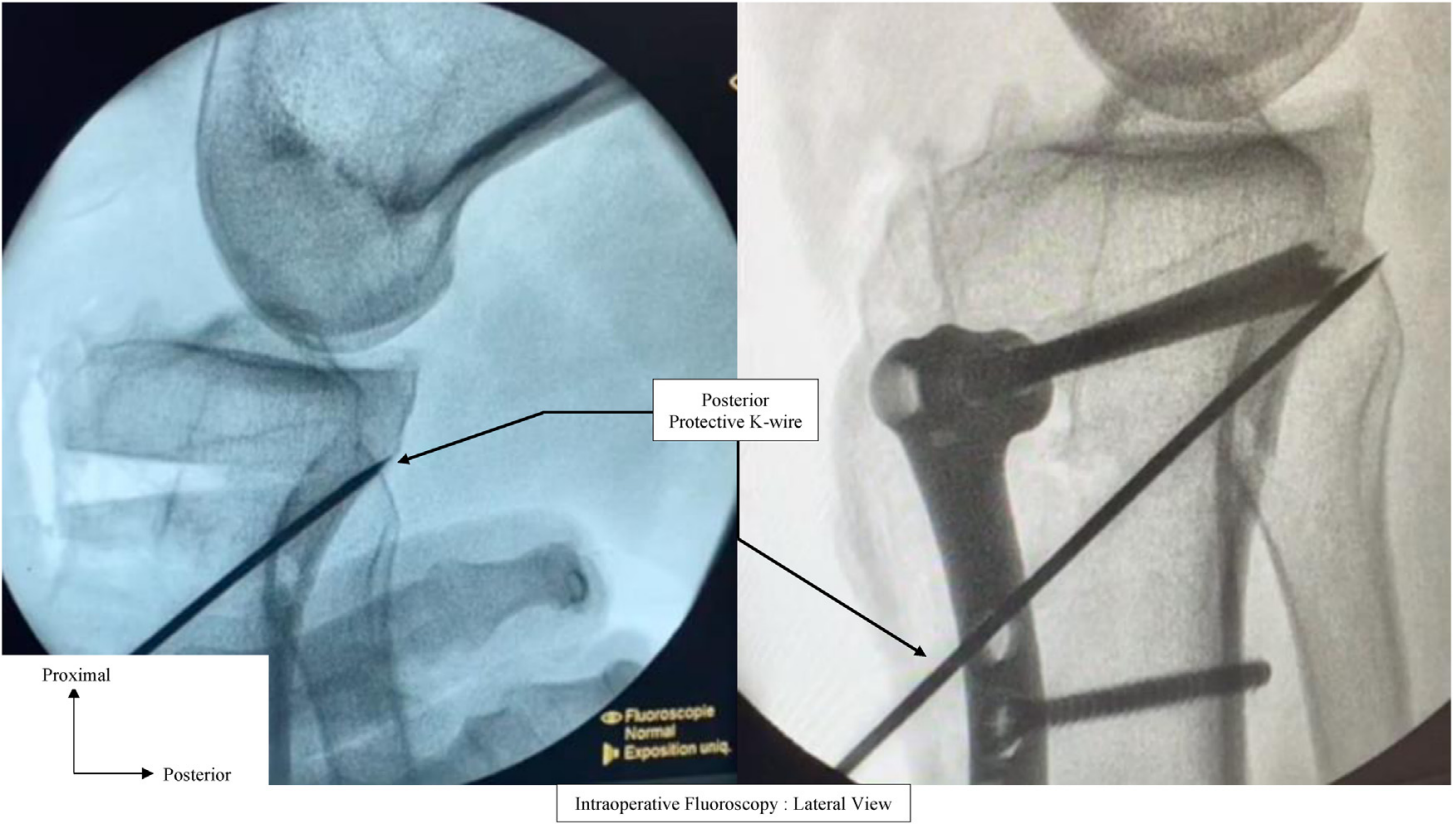
Intraoperative fluoroscopy. The posterior protective K-wires are left in place during reduction and osteosynthesis to prevent posterior hinge fracture. Left: Lateral view after removal of the anterior bone wedge. Right: Lateral view after reduction and plate osteosynthesis.

Once all the cuts are done, all k-wires are removed except the 2 hinge K-wires and the 2 PSCG are removed from the proximal tibia. The anterior bone wedge is then removed, and reduction is achieved by extending the knee. The plate is positioned on the anteromedial tibial in line with the predrilled holes. Perfect correlation of the plate screw holes and predrilled holes in the tibia confirms the desired correction in the sagittal and frontal plane (PTS and medial proximal tibial angle) (Fig 5).

### Rehabilitation

Early rehabilitation focuses on restoration of full extension and quadriceps activation exercises. Patients are placed in a posterior cruciate ligament (PCL)-protecting brace, avoiding hyperextension, and thus decreasing the risk of postoperative excessive recurvatum.

Weight-bearing is partial and protected by crutches until the sixth week. Radiologic evaluation of the osteotomy is then performed to allow patients to increase the weight-bearing gradually. Open-chain muscle strengthening is contraindicated until 3 months postoperatively. Cycling is allowed at 6 weeks, running and nonpivoting sports at 4 months, pivoting noncontact sports at 6 months, and pivoting contact sports at 8 to 9 months.

## Discussion

The present technique describes a reliable and reproducible retro-TT ACW-HTO using PSCGs. PSCGs may facilitate less-experienced surgeons in performing this complex surgery and aid in the learning curve. In addition to decreasing the posterior tibial slope, corrections in the frontal plane also may be required. The combination of a slope and varus correction HTO decreases anterior tibial translation and ACL forces more than either one individually.^11^ Three-dimensional preoperative planning could help the surgeon to conceive biplanar osteotomy with asymmetric anterior closure. A posteromedial opening wedge-HTO could also be performed when metaphyseal tibial varus deformity is diagnosed,^22^ but a MOW-HTO will be more restrictive in correcting the slope just as an ACW-HTO in correcting the varus.

Although the use of PSCG could help to achieve accurate correction in line with the pre-operative planning,^19^^,^^20^ the ideal postoperative slope target is not clearly defined in the literature. It is accepted that postoperative PTS should not exceed the threshold of 12°.^5^, ^6^, ^7^ There is, however, a proportional increase in the risk of ACL graft failure^6^ and pathologic anterior tibial translation^8^ as the slope increases. Shelbourne et al.^23^ also found an increased risk of subsequent graft tears after primary ACLR in case of PTS exceeding 10°. Careful consideration should be made to not excessively reduce the slope as to PCL stress,^24^ thereby risk of native PCL injury^25^ and PCL reconstruction graft laxity.^26^ Slope-correcting osteotomy can also lead to postoperative recurvatum, which is also identified as a risk factor for ACL graft rupture if greater than 5°.^27^ Pangaud et al.^21^ estimated the mean value of PTS to be 6.3° based on 378 CT scans of healthy participants, this being therefore considered by the authors as the postoperative goal (PTS between 5 and 7°). A postoperative recurvatum PCL brace was routinely used to prevent recurvatum in the present technique.

The preservation of the TT during slope change osteotomy remains a question of interest. Clinical relevance has not been established, as similar clinical outcomes were observed whether TT osteotomy is performed or kept intact.^12^, ^13^, ^14^^,^^28^ TT osteotomy, however, exposes the patient to specific complications such as delayed union or hardware-related pain.^29^ Better control of the patellar height may be achieved when performing a TT osteotomy during slope changing osteotomy.^30^ While there is extensive literature regarding patellar height and OCW-HTO,^31^, ^32^, ^33^, ^34^ no study has yet focused on this issue in ACW-HTO with TT preservation. Dejour et al.^14^ found no significant change in patellar height after retro-TT ACW-HTO; this was a small study involving a subanalysis of a clinical study involving 9 patients. Measurement of patellar height using traditional indexes could also be biased by the slope correction osteotomy. Thus, the Blackburne-Peel index has been proven to be directly affected by PTS.^35^ Described in 2017, the femoral patellar height index is a femur-referenced patellar height measurement method avoiding HTO-related alterations of the proximal tibia.^36^ This index could be useful to accurately measure the impact of ACW-HTO on patellar height. Pending future investigations, it is the authors’ opinion that the benefit of preserving the integrity of the extensor mechanism outweighs the potential risk of patellar height change.

There are limited data regarding the clinical outcomes of ACW-HTO combined with ACLR. Only a few clinical series have been published.^12^, ^13^, ^14^^,^^28^^,^^37^ No ACL graft failure was reported at a minimum of 2 years’ follow-up, and good clinical outcomes were achieved with respect to anteroposterior stability and patient-reported outcome measures. The main limitation of these studies was their limited sample size, with the largest one including 22 patients.^13^ This makes it difficult to achieve a consensus on the indication for ACW-HTO, given the limited evidence available. Song et al.^28^ suggest that slope-changing osteotomy should be performed along with primary ACLR in cases of slope steeper than 13°, excessive anterior tibial subluxation in extension, and chronic meniscus posterior horn tears. Other authors, however, recommend ACW-HTO at the first^13^ or second^12^^,^^14^ revision ACLR for PTS greater than 12°. The authors’ opinion is in line with Dejour et al.^14^ and Sonnery-Cottet et al.,^12^ as this technique is performed only in case of a second revision ACLR or more.

The main disadvantage of this technique is the risk of injury to the popliteal artery. It can be minimized using peroperative fluoroscopy and protective K-wires ahead of the posterior hinge. The maximum saw length to be driven into the tibia can also be estimated with the help of the preoperative CT scan. Advantages and disadvantages are highlighted in Table 3.

**Table 3:** Advantages and Disadvantages of the Present Technique

Advantages	Disadvantages
• Easier learning curve • Increased accuracy and reproducibility • Easier planning of biplanar HTO • Reduced risk of ACL tunnel convergence • Reduced risk of TT fracture • Reduced risk of posterior hinge fracture	• Risk of injury to the popliteal artery • Risk of injury to the infrapatellar branch of the saphenous nerve • Risk of injury to the common fibular nerve • Hardware-related pain • Preoperative CT scan irradiation

ACL, anterior cruciate ligament; CT, computed tomography; HTO, high tibial osteotomy; TT, tibial tubercle.

## Supplementary Data

ICMJE author disclosure forms

Video 1 Retro-tibial tubercule anterior closing wedge-high tibial osteotomy using a patient-specific cutting guide surgical technique for a 31-year-old male patient with second anterior cruciate ligament rerupture and posterior tibial slope exceeding 12°.
